# Barriers to delivering mental health services in Georgia with an economic and financial focus: informing policy and acting on evidence

**DOI:** 10.1186/s12913-018-2912-5

**Published:** 2018-02-13

**Authors:** Lela Sulaberidze, Stuart Green, Ivdity Chikovani, Maia Uchaneishvili, George Gotsadze

**Affiliations:** 1 Curatio International Foundation, 3 Kavsadze str, 0179 Tbilisi, Georgia; 2 National Institute for Health Research Collaboration for Leadership in Applied Research and Care, Northwest London, Imperial College London, London, UK

**Keywords:** Mental health, Health systems, Health policy, Georgia, Low-and-middle income countries

## Abstract

**Background:**

Whilst there is recognition that the global burden of disease associated with mental health disorders is significant, the economic resources available, especially in Low and Middle Income Countries, are particularly scarce. Identifying the economic (system) and financial (individual) barriers to delivering mental health services and assessing the opportunities for reform can support the development of strategies for change.

**Methods:**

A mixed methods study was developed, which engaged with a range of stakeholders from mental health services, including key informants, service managers, healthcare professional and patients and their care-takers. Data generated from interviews and focus groups were analysed using an existing framework that outlines a range of economic and financial barriers to improving mental health practice. In addition, the study utilised health financing and programmatic data.

**Results:**

The analysis identified a variety of local economic barriers, including: the inhibition of the diversification of the mental health workforce and services due to inflexible resources; the variable and limited provision of services across the country; and the absence of mechanisms to assess the delivery and quality of existing services. The main financial barriers identified were related to out-of pocket payments for purchasing high quality medications and transportation to access mental health services.

**Conclusions:**

Whilst scarcity of financial resources exists in Georgia, as in many other countries, there are clear opportunities to improve the effectiveness of the current mental health programme. Addressing system-wide barriers could enable the delivery of services that aim to meet the needs of patients. The use of existing data to assess the implementation of the mental health programme offers opportunities to benchmark and improve services and to support the appropriate commissioning and reconfiguration of services.

**Electronic supplementary material:**

The online version of this article (10.1186/s12913-018-2912-5) contains supplementary material, which is available to authorized users.

## Background

Mental health, substance use, and other behavioral problems characterize all nations. These disorders have become increasingly significant contributors to the global burden of disease over time. The burden of disease associated with mental and substance use disorders in 1990 was estimated at 5.4% of all global disability-adjusted life years (DALYs). This proportion increased to 7.4% in 2010, which constitutes a 37% increase over the last two decades [1, 2]. Mental health disorders represent the largest single part of the global economic burden worldwide. It has been estimated that these conditions will account for the loss of an additional 16.1 trilion US Dollars by the end of 2030 [3]. Despite the significant prevalence of mental health disorders and their global economic burden, the average proportion of national health budgets allocated to mental health in low and middle income countries (LMICs) is estimated to be less than 2% [4, 5]. While the need for access to mental health services may be greater in LMICs, the availability of resources is disproportionately lower than in high income countries. Consequently, the ability of countries with scarce resources, such as Georgia, to efficiently deliver services, is paramount.

### An overview of the Georgian mental health system

Georgia is a Former Soviet Union (FSU) country in the Caucasus region, with a population of 3.7 million people. Georgia has transformed healthcare financing and has undertaken a number of health system reforms over the last two decades, including in the mental health sector [6, 7]. The *Universal Health Coverage Programme* (*UHCP*), a state funded programme introduced in 2013, mainly provides primary care for individuals with physical health problems, but includes some limited mental health disorders [8]. Specialist in-patient and outpatient psychiatric services are covered separately by the *State Programme for Mental Health* (*SPMH),* which was introduced in 1995 and managed by the Social Service Agency as part of the Ministry of Labour, Health and Social Affairs (MoLHSA) [9]. As such, outpatient and acute and long-term inpatient psychiatric care is available free of charge to all citizens of Georgia through 23 mental health services distributed across the country. Services include outpatient consultations with a psychiatrist, and subsequent prescriptions, either in one of 10 independent ambulatory centres or polyclinics, or in one of eight psychiatric hospitals that have an established outpatient department. Outpatient care also covers psychosocial rehabilitation services, which are offered at 2 outpatient centres and 1 independent facility, and psychiatric crisis resolution, which is provided at 4 hospitals with outpatient services. Currently, a number of common mental disorders, such as anxiety and obsessive-compulsive disorders (OCD), are excluded from any outpatient treatment.

In-patient care includes a broader range of services compared to outpatient care, and whilst most inpatient care is provided free, care provided for alcohol-related psychiatric disorders are subject to a sizable co-payment [7]. Care is also provided to those living in supported housing (a combination of accommodation and support services to maintain independent living). There are 9 standalone hospitals throughout the county and 3 inpatient psychiatric units integrated into the general hospitals in the capital city, which only provide acute inpatient care services. The reallocation of psychiatric hospital beds from large institutions to newly opened psychiatric departments within general hospitals in 2011 was seen as one of the most significant reforms within the mental health system in Georgia [7].

The financing mechanisms for delivering services differ according to the type and setting of the services provided. Outpatient services are financed directly through the historic allocation of funds from the *SPMH* budget, whilst long-term inpatient services are reimbursed through a standard per-diem or monthly tariff. The exception is for alcohol-related disorders where case-based payment is used. Although there is much discussion about modernising the delivery of mental health services to ensure equity of access to treatment, there are no studies documenting barriers to care [7]. The only study among the Internally Displaced Population found that utilization of psychiatric services for common mental disorders is unsatisfactorily low and one of the major barriers in service utilization are costs related to drugs and services [10].

In this regard, this study utilizes the country of Georgia as a case study in order to achieve following research objectives: 1) Describe the challenges in delivering mental health services in Georgia; 2) Identify the role that financial and economic barriers play in the current and future allocation of such resources.

It is hoped that the evidence from the study will inform the national mental health policy debate to improve the quality of services provided for those with mental health, substance misuse and other behavioural problems.

## Methods

### Study design

During the first phase, a desk review was carried out of the national mental health programme, relevant financial documents and public database. The study was designed using qualitative research methods, including the use of in-depth interviews and focus groups discussions (FGDs).

### Theoretical framework

A desk review of the existing literature on economic and financial barriers to mental health programmes enabled the design of a framework to support qualitative data collection and analysis. A topic guide was developed by the investigators based on a theoretical framework proposed for mental health research by Knapp et al. in LMICs, as shown in Table 1 [11]. The topic guide (please see Additional file 1) was broad enough to elicit a wide range of views from study participants, and yet specific enough to ensure alignment with the framework.

**Table 1:** Theoretical framework of economic barriers [11]

Information barriers	Resource inappropriateness
• Limited evidence base • Difficulties of transferring services research findings	• Services do not match the needs • Dominance of large institutions • Over-investment in expensive technology
Insufficiency of resources	Resource inflexibility
• Poor economic conditions • Vulnerable currency • Low willingness/ability to pay • Poor stewardship	• Centralized budgets • “Benefit trap” disincentives • Poor coordination across agencies • “Silo budgeting”
Resource distribution	Resource timing
• Concentration in urban areas • Highly institutionalized services • Neglect of particular disorders	• Supply inelasticity • Training delays • Capacity-constrained systems

### Data sources

#### Desk review exercise

Quantitative data were gathered from the MoLHSA including programmatic data, National Health Accounts, which report the financial expenditure for the *SPMH,* from a mental health atlas and from the European Health for All database (HFADB) [12].

Programmatic data from the MoLHSA were requested in order to reveal existing differences in spending and mental health workforce between different mental health institutions. Data collated from central government documents were used to identify the range of services provided across the country and to compare the capacity, diversity and resources associated with these services.

In order to ascertain current trends in health expenditure and compare Georgia to other countries, data were obtained from the HFADB and the mental health atlas. The number of metrics identified, with sufficient data for comparison, included:

1. Total health expenditure as % of Gross Domestic Product (GDP);
2. Total health expenditure, dollars using Purchasing Power Parity (PPP) rates;
3. Mental health government expenditure as % of total health expenditure; and
4. Mental hospital expenditure as % of the total mental health budget.

The metrics for Georgia were compared with aggregates of EUR-A, EUR-B, EUR-C group countries and Commonwealth of Independent States (CIS) countries [as defined by the World Health Organization (WHO)], and the median values for lower middle income (LMC), upper middle income (UMC) and high income countries (HIC), as defined by the World Bank criteria.

#### Qualitative data collection

Data were generated from in-depth semi-structured interviews and FGDs. Forty-one face-to-face semi-structured interviews were held with a range of stakeholders in order to obtain comprehensive information and attain rigorous study findings (Table 2). Key informants included policy-makers; representatives of non-governmental organizations (NGOs); and service managers and psychiatrists from various mental health facilities, including outpatient departments, rehabilitation centres and acute/long-term psychiatric hospitals. In addition, interviews were conducted with a number of patients and their caretakers. Following the in-depth interviews, two FGDs were held with psychiatrists representing various mental health facilities from across the country; one FGD was specifically held for staff from outpatient facilities and the other for staff from inpatient facilities. Table 2 provides details about the study respondents.

**Table 2:** An overview of the data collection methods, respondent type and number of participants

Type of Respondents	Number of Respondents	Method
Key informants	7	In-depth interview
Policy-makers	4	
Representatives from NGOs	2	
Managers of the service provider facilities		In-depth interview
Out-patient facility	1 (out of 10)	
Independent rehabilitation centre	1 (out of 1)	
Out-patient & Rehabilitation	1 (out of 2)	
Out-patient & Hospital	6 (out of 8)	
Acute inpatient care facility	2 (out of 3)	
Hospital (long-term care)	1 (out of 1)	
Psychiatrists		In-depth interview
Out-patient facility	3	
Inpatient facility	3	
Caretakers	7	In-depth interview
Patients	9	In-depth interview
Psychiatrists		Focus group discussion
Out-patient facility	6	
Inpatient facility	4	

The interviews were conducted by researchers, recorded and transcribed *verbatim* by research assistants, and subsequently validated by the interviewers. Written or verbal consent was sought from all participants at the time of the interview. The FGDs were led by a researcher and recorded and transcribed *verbatim* by research assistants and validated by researchers.

#### Qualitative data analysis

The theoretical framework that described economic and financial barriers was utilised to inform the qualitative data collection; it was also used as a basis for the analytical framework [11]. The analytical framework was systematically applied to code and analyse the data deductively; consideration was also made to identify emergent themes that were not coded within the analytical framework. The two researchers coded all transcripts by hand independently and incorporated all findings into a comparative table. The table was shared with the research team prior to the discussion meeting set for the final agreement. Some exisiting inconsistencies and disagreements between the two researchers concerning initial coding was solved at the meeting and a final consensus was achieved.

## Results

### Desk review findings

**Fig. 1:**
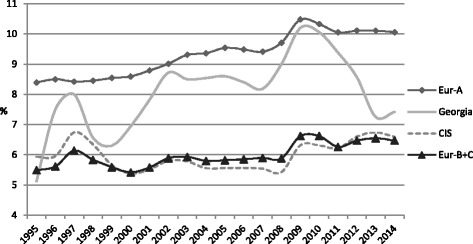
Total Health expenditure as percentage of GDP (1995–2014) [12]

Health expenditure data showed that Georgia has seen a substantial increase in the proportion of GDP spent on healthcare since 1995 (Fig. 1), with an 83% increase between 1995 and 2011. This was similar to EUR-A group countries, where increasing health expenditure trends are also seen. Expenditure decreased rapidly in the period 2009–2013. Even so, in 2014 Georgia’s health expenditure as a proportion of GDP stood at 7.4%, which was still greater than CIS and EUR B + C countries.

**Fig. 2:**
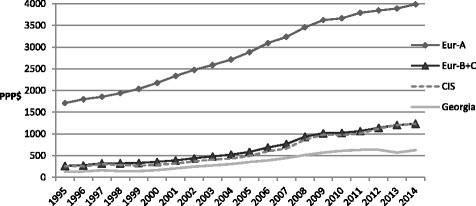
Total health expenditure, PPP$ per capita (1995–2014) [12]

An analysis of per capita total health expenditure in PPP $ (Fig. 2) shows that, although the proportion of GDP spent on health was relatively high in Georgia, actual health expenditure (PPP $) was lower than in the other groups.

**Fig. 3:**
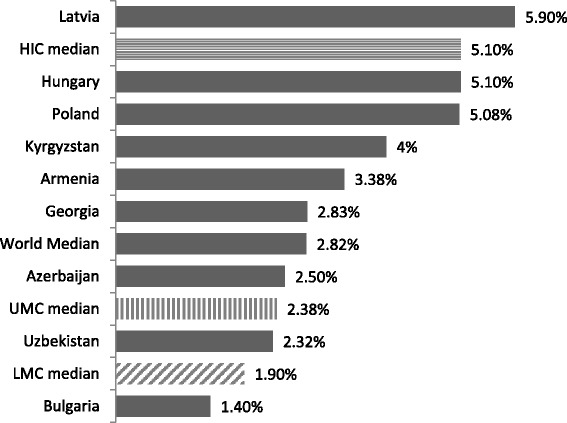
Mental health government expenditure as a percentage of total health expenditure, 2011 [5]

Mental health expenditure as a percentage of total government health expenditure was compared between a number of countries (Fig. 3). Whilst Georgia’s mental health expenditure of 2.83% of total health expenditure is higher than the 2.32% median mental health expenditure of UMCs, this is still nearly half the amount of the median value for HICs at 5.1%, indicating that Georgia has a long way to go.

**Fig. 4:**
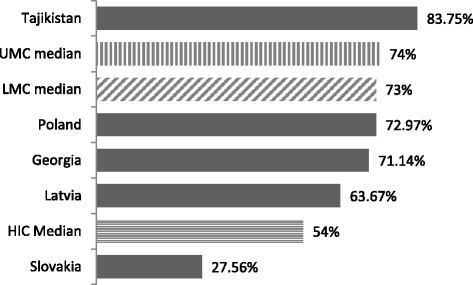
Mental hospital expenditure as a percentage of total mental health expenditure, 2011 [5]

An analysis of the distribution of resources between outpatient and in-patient care was further undertaken through comparing expenditure on mental hospitals as a percentage of the total mental health spending for 2011 (Fig. 4). It can be seen that this distribution varies greatly across countries. Georgia spent 71.14% of its total mental health budget on inpatient care in 2011, a value similar to the median of other lower middle-income countries and UMCs (73% and 74%, respectively). However, an almost equal distribution of resources between inpatient and outpatient care is observed in HICs, with a median of 54% allocated to inpatient care.

Due to limited data availability, further detailed analysis of international comparators was not possible. However, local data allowed some comparison between different mental health institutions within Georgia. Analysis revealed that there are twenty-three individual facilities that offer a range of services from long-term in-patient care to supported accommodation. Across these facilities, 18 offer outpatient services; 12 offer long-term in-patient services with a capacity of 1207 beds; 10 offer acute in-patient services; four provide crisis intervention; three provide psycho-rehabilitation; and only one provides supported accommodation (Table 3). The total number of psychiatrists working across these services was estimated at 176 (3.92 psychiatrists per 100,000 people). In addition to the services on offer, Table 3 also demonstrates the huge variations between regions in terms of the availability of services. Due to the extreme topographical nature of Georgia, straight-line distances do not accurately reflect the true distance between populations and services. While services are, as one would expect, most often located in the populous cities of the region, some regions are still left underserved.

**Table 3:** Prevalence of patients with a mental disorders and availability of mental health resources by region (2015)

Region	Population size (in thousands)	Prevalence of mental disorders per 100,000 population	Facilities offering mental health services	Outpatient mental health clinics	Long-term care facilities (beds per 100,000 population)	Acute mental health departments	Crisis intervention service	Psycho-social rehabilitation services	Supported housing	Psychiatrists per 100,000 population
Tbilisi	1111.0	1294.0	7	3	26	5	1	1	0	6
Adjara	335.7	4408.1	1	1	4	1	1	0	0	3
Guria	113.1	3037.2	2	2	0	0	0	0	0	2
Imereti	533.2	3616.3	3	3	90	2	1	1	1	6
Kakheti	318.4	6057	2	2	0	0	0	1	0	3
Mtskheta-Mtianeti	94.4	1913.1	1	1	0	0	0	0	0	2
Samegrelo-Zemo Svaneti	330.1	2755.5	2	2	3	0	0	0	0	2
Samtskhe-Javakheti	160.6	1389.8	1	1	0	0	0	0	0	1
Kvemo Kartli	425.3	1337.2	2	1	27	1	1	0	0	2
Shida Kartli	263.6	3208.3	2	2	35	1	0	0	0	3
Racha-Lechkhumi and Kvemo Svaneti	31.7	–	0	0	0	0	0	0	0	0
Total	3717.1	2327.0	23	18	27	10	4	3	1	4

### Qualitative data findings

**Fig. 5:**
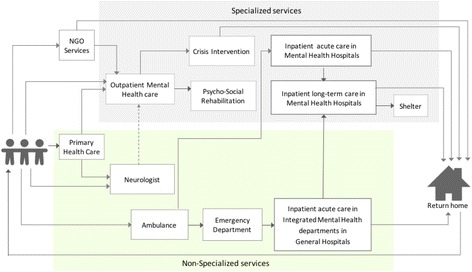
Patient-pathway demonstrating access points to inpatient and outpatient care and the connection with additional services

Through the qualitative analysis, a typical patient pathway within the mental health system in Georgia was developed. This is used as a framework for reporting the findings of the study to better contextualise them in terms of patient access to services (Fig. 5). Barriers are reported at the patient/organisational (micro/meso) level from both the outpatient and inpatient care perspectives as well as at the higher system (macro) level.

### Micro-meso: Outpatient level barriers in mental healthcare

The study identified a number of barriers at the patient/organisational level from an outpatient care perspective. One of the most significant barriers within outpatient care is that of initial diagnosis and appropriate referral, which, although not directly related to financial or economic issues, has a profound impact on the delivery of, and access to, appropriate services. In most cases, people with mental health problems initially attend primary care where they are assessed by a family physician. Following diagnosis of a mental health disorder (following ICD-10), family physicians are only authorised to manage mild/moderate depression, including the prescription of anti-depressants. In practice, patients usually are referred to neurologists, rather than psychiatrists, for further evaluation and treatment. There is a general lack of awareness among family physicians regarding the exact competencies of psychiatrists, compared with neurologists, with whom they are often more familiar. The geographical location of psychiatrists also plays a role: neurologists are often co-located in the same facility as family physicians, whilst psychiatrists are located in separate psychiatric facilities.

Although family physicians have a gate-keeping role in the Georgian health system, often patients self-refer to neurologists, usually requiring out-of-pocket expenses for consultations and medications that would otherwise be free as part of the *SPMH,* if treatment were sought from psychiatrists. In addition to the cost for patients seeking treatment from neurologists, many psychiatrists are concerned that the management and treatment of mental health disorders by neurologists is often sub-optimal.

In specialised outpatient mental health facilities, treatment including medication is provided free of charge. Patients usually attend outpatient services following discharge from psychiatric inpatient care from either a specialist facility or a general hospital, and rarely through direct referrals from family physicians. In addition, the distribution of resources to a facility based on historic allocation and not based on the number of patients or the population of their catchment area results in an allocative inefficiency. This study identified an unequal distribution of the *SPMH* budget among outpatient facilities. For example in 2013, the annual per capita outpatient program cost ranged from 4 Georgian Lari (GEL) (2.5 USD) to 17 GEL (10.6 USD) depending on outpatient facilities.

*“…The global budget was estimated based on the registered patients in outpatient facilities a very long time ago. Nowadays some of these facilities serve a greater amount of patients than they used to do and vice versa, but nothing has changed in the amount of money we receive from the Ministry…” – A mental health facility manager*

Outpatient facility managers complain about an insufficiency of resources that results in the acquisition of low-cost drugs or a shortage of drugs. In these cases, where possible, patients are encouraged to purchase their own medications elsewhere, or patients may choose to do so as they perceive the quality of the drugs provided to be poor. This effectively contradicts the ‘free provision of medications’ stated by the *SPMH* and poses a financial barrier for patients due to out-of-pocket payments.

*“… At the end of the month, I sometimes experience a shortage of drugs for my patients. In that case I have to ask the Ministry for an adjustment, but it takes time and the patient is forced to buy the drugs on their own…” – A outpatient clinic manager*

*“…The psychiatrist [privately] prescribed better quality drugs, but they are expensive and I cannot always buy them…” – A patient*

Furthermore, there is a perceived lack of knowledge related to public procurement procedures among mental health facility staff members. Whilst many facility managers complain that the regulations for public procurement are restrictive and prohibit the purchase of higher quality medications, others suggest that in fact the flexibility of the regulations actually facilitates the purchase of higher quality medications.

Appropriate staffing of facilities was also identified as an issue by the study. Whilst the *SPMH* stipulates that multi-disciplinary care should be provided for patients by healthcare professionals, most outpatient clinics are staffed solely by psychiatrists and nurses.

The study revealed further geographical and financial barriers to appropriate access to treatment. Continuous follow-up, especially for the collection of medication, often requires monthly appointments at mental health outpatient departments, meaning that patients often have to travel long distances, which can be prohibitive due to the costs/time involved.

*“…It’s difficult for me to afford a transportation fee. Today my neighbour took me here (in the mental health facility) in his car…” – A patient*

In addition to access, another financial barrier includes informal out-of-pocket payments, which could be due to the legacy of previous regulations that levied a preliminary charge for an initial consultation with a physician. Although no longer obligatory, some providers continue to request this payment.

*“…When I have a visit to the psychiatrist, she gives me 10 pills for free. But I’d rather buy 100 pills for 7 GEL [4.5 USD] rather than visit the psychiatrist and pay more for that visit. As a rule, I have to make a 40 GEL [25 USD] direct payment for this visit…” – A patient’s care-taker*

Outpatient care also includes crisis intervention and psycho-social rehabilitation services. Whilst crisis services receive a significant proportion of the outpatient budget, accounting for 20% across four centres, which enables them to provide good quality of care, medications and laboratory investigations, the demand far outstrips the provision of services.

The provision of psychosocial rehabilitation is limited as well: only three facilities offer this service nationally, and since the average duration for each patient is six months, the result is relatively long waiting times.

### Micro-meso: Inpatient level barriers in mental healthcare

The route of entry as an in-patient in a mental health department is usually through an outpatient clinic, crisis intervention centre or via emergency services. On admission, most patients remain in the acute department for 2–3 weeks. During this period, the care and medications provided at a general hospital are often perceived to be of higher quality than elsewhere, and patients are able to access a wider range of services, diagnostic investigations and referral to various (non-mental health) specialists.

*“...I have enough resources for my patients to provide them various high quality investigations: I can use the computer tomography procedure twice if needed during the admission period. This is not the case for long-term care psychiatric hospitals. Medications are also of higher quality, with fewer side-effects….” - An acute care facility manager*

As such, there is a high demand for acute in-patient services in general hospitals, often resulting in a lack of available beds and subsequent waiting periods for patients. During the mental health reforms in 2011, when there was significant deinstitutionalization of hospital care and integration of psychiatric beds into general hospitals, the average cost of care was estimated to determine a tariff for acute inpatient care within an agreed period of hospitalisation. A tariff of 840 GEL (525 USD) per case, with a maximum stay of 2–3 weeks, was deemed acceptable/sufficient for private hospitals to provide such services. Since the monthly voucher for long-term in-patient care is equal to 450 GEL (281 USD), in-patient facilities are motivated to fully absorb this tariff, so all patients are retained in acute care departments for 2–3 weeks. Following this period of acute care, patients are either discharged home with a recommendation to continue out-patient treatment, or the patient is transferred to ‘long-term’ in-patient care. Since there is no formalised continuity between in-patient and out-patient care, clinical information and medical records are not shared, which often means that if patients do not attend an out-patient appointment following their admission there is no system to identify this. This is further exacerbated by the lack of community services for psychiatric patients, which often results in deteriorating mental health and the subsequent rehospitalisation of patients.

In an effort to reduce hospital readmission, the MoLHSA established a 7-day readmission period; if a patient is readmitted within this period the payment to a provider is withheld, unless the case is investigated to identify reasons for the readmission, which can take several months. Those working within the facilities report considerable dissatisfaction with this policy, and as such often manipulate data to avoid penalties.

*“…The seven day readmission period was inappropriately chosen for psychiatry for the following reasons: first, there is fragmented mental health care, second we have not developed community services, and finally families/society are not educated well enough to take appropriate care for such patients. That’s why discharged patients often need re-admission…” - A psychiatrist*

Those patients who are not stabilised and ready for discharge within the 2–3 week period of acute inpatient care are transferred to long-term care for continued inpatient treatment. Considering that only 100 places are available in supported accommodation in the whole country, and that there is no other provision for residential services, many patients end up in long-term psychiatric facilities unnecessarily, creating a shortage for those patients that clinically require these services.

*“…There is a need to develop residential services for mental health patients who are discharged from in-patient care. For example, I have many patients in the long-term care department who have lived there for more than 3 years. There are no places for them outside psychiatric hospitals helping them to live in their own way*…” *- A mental health facility manager*

A further factor that might influence the retention of acute patients in long-term care is the perverse incentive to maintain full occupancy in long-term facilities as payments are provided per capita. Despite this, there are often significant shortages of clinical staff, especially psychiatrists, within the long-term care facilities.

In addition, the workload for psychiatrists may be seen as overwhelming due to the significant amounts of paper work.

*“… I am a psychiatrist in this hospital and serve 65 patients per night. It is really difficult for me to pay adequate attention to patients, because preparing the documentation takes most of my time…” - A psychiatrist*

A further barrier for patients in accessing appropriate care, especially in psychiatric facilities, is the difficulty of referral to other medical specialties for physical health care issues. This has been attributed to strict policies that state that patients cannot be in receipt of more than one state-funded health programme simultaneously. In practice, this means that patients will often have to wait to be discharged from *SPMH*-funded care before they may access other healthcare services.

### Macro: System/policy level barriers to mental healthcare

The study identified the following barriers at the system level that affect the delivery of services. These include information barriers, poor stewardship, resource distribution and resource inflexibility.

A significant challenge in implementing the *SPMH* is the lack of any mechanism to monitor and evaluate programme delivery and effectiveness. Whilst routine administrative data are collected electronically, this is not used for any purpose other than the remuneration of facilities. As such, policy-makers do not have ready-to-use information to assess the relative effectiveness of different treatment/service models in a way that might inform decision-making. In addition, there are no systems to assess and monitor the quality of care delivered by facilities funded through the *SPMH,* as there are no national, or even local, quality indicators for mental health systems in Georgia.

*“…With the 7 days re-admission rate, we are not able to assess the quality of care. In my opinion, it was not worth incorporating this in the SPMH at this stage…” - A policy maker*

This is also related to the lack of human resources at the policy level, with just a single person responsible for the collection of data from mental health facilities and acting as an interface between the delivery of the *SPMH* and ministerial oversight. Whilst there is a relatively sophisticated system for collecting data submitted by mental health facilities, weak technical capacity means that the data cannot used for decision-making.

At the facility level, managers often complain that they do not have the autonomy to re-appropriate *SPMH* funding based on clinical need, due to strictly enforced “silo budgeting” and strict public finance management rules which prohibit resource re-allocation within the different components of the program. For example, in cases where there is a shortage of medications in an outpatient department but a surplus within the inpatient department of the same facility, it is impossible to transfer medications between the two due to restrictive policies.

Some common mental health disorders such as anxiety and OCD are not covered by the *SPMH*, and outpatient facilities are restricted to deliver services to the mental health patients. This often results either in out-of-pocket payments by the patients to receive services, or in psychiatrists manipulating the diagnosis in order to comply with the regulations to deliver the services needed.

A lack of vision and strategy, coupled with inadequate investment in the mental healthcare system, especially in terms of work-force development and capacity building, has led to substantial losses in staff. Additionally, low salaries and perceived difficult working conditions ensure that psychiatry remains an unattractive choice of specialty for medical students. In addition to the shortage of psychiatrists, there is also a shortage of psychologists, nurses and social workers throughout the country with no current plans or policies underway to build capacity and develop the necessary workforce.

*“… The educational level among nurses is not enough; they study for a three-year period but have little awareness of psychiatry. Also the knowledge of psychiatrists is out of date, as there are no trainings or conferences that would help improve our qualifications, especially those working in more remote regions …” - A psychiatrist/facility manager*

## Discussion

The results present a broad range of barriers to the development and implementation of reforms within the current mental health system in Georgia. These barriers are presented at the micro-meso and macro level aligned to the patient pathway to provide an overview of the delivery of mental health services. The barriers were identified using the framework developed by Knapp et al. and are further contextualised here using this framework [11]. The framework proposes six individual resource barriers, defined as barriers that negatively affect the incidence, treatment or impact of mental health disorders due to inefficiencies and inequities in the use of resources. These barriers include information barriers, insufficiency of resources, resource distribution, resource inappropriateness, resource inflexibility and resource timing [11].

### Information barriers

Information barriers were broadly defined by Knapp et al. to include the translation of research evidence into service development [11]. Georgia, like many LMCs, has a limited capacity for undertaking in-country research activities. Therefore, this barrier was interpreted more broadly.

At a patient level, there is a lack of information about the free treatment available for those with mental health conditions. The absence of explicit policies or guidelines for the consistent identification and on-ward referral to mental health services following a diagnosis of a mental health disorder propagates the ineffective and costly (to the patient) treatment of patients.

Information barriers at the system level include barriers to the transmission of information about best practice at a clinician/organisational level, but also the lack of local evidence available to policy-makers about the effectiveness of the *SPMH*. Furthermore, whilst there are relatively sophisticated systems in place for collecting data about patients and their treatment through electronic patient records, there is no overall monitoring of services to identify the most effective care models/pathways that could be used in the planning and commissioning of services by policy-makers. This is further highlighted by the lack of any quality measures or indicators that allow benchmarking of services and opportunities for transparent oversight and scrutiny of the delivery of services, which are reported as being highly variable. In addition to this, the lack of awareness of public procurement procedures among facility managers is one of the barriers that leads to low quality drugs procurement at outpatient mental health facilities.

### Insufficiency of resources

A major problem facing many LMICs is that of allocative planning for mental health. The majority of LMICs, especially those in Africa and Asia, spend less than 1% of their total health budget on mental health [13]. Lower middle-income countries, such as Georgia and some other FSU countries, spend an average of 2.62% of their total health budget on mental health, compared with higher-middle-income countries and high-income countries, which spend 4.27% and 6.88% respectively. As demonstrated by the logarithmic scale for the relationship between the budget for mental health (as a proportion of total health budget) and GDP, the poorer the country the less is spent on mental health [13]. Knapp et al. identified the following reasons for resource insufficiency: poor economic conditions; the low priority attached to mental health by the government or other key funders; low willingness to seek or pay for treatment; and poor stewardship [11].

In terms of prioritisation by the government, the picture is mixed. Following the introduction of the *SPMH,* mental health services and medications have been provided free at the point of care, addressing one of the major barriers to improving mental healthcare. Although healthcare expenditure in Georgia has increased, the allocation of funding for mental health has only seen a modest rise, which is insufficient to deliver effective and efficient services. This is exemplified by the low quality of medications provided by many facilities, where patients often prefer to purchase their medications privately, outside the *SPMH*, to access higher-quality medications. The existence of independent procurement practices and restricted budgets at each facility does not allow the purchase of high quality drugs at a lower price. A unified procurement mechanism might solve this problem. Furthermore, due to their high workload, psychiatrists are restricted in the amount of time available for consultations, limiting the possibility of delivering psychological therapies. Nevertheless, even with limited resources, there seems to be potential to improve allocative and technical efficiency by better integrating services and setting standards to improve the quality of drugs.

### Resource distribution

In Georgia, the highest population density is seen in the capital, Tbilisi, which has the highest number of acute facilities (five) but a relatively small number of long-term acute beds. Conversely, the Racha-Lechkhumi & Kvemo Svaneti region, a particularly remote and sparsely populated rural area, has no provision of mental health services. As a result, patients are often required to travel large distances. This can be prohibitive due to the costs/time involved and creates a barrier to accessing treatment. As there are currently no reliable data on the prevalence of mental disorders across the regions of Georgia, it is extremely difficult to effectively plan services that meet the needs of the population. Instead, budgetary distributions to facilities are based on historic allocations. This highlights a particular problem faced by those proposing reform of mental health services. In order to develop a comprehensive mental health policy and subsequent programme, a needs-based policy assessment is required [14]. This would necessitate a more rigorous understanding of the needs of patients through epidemiological surveys of the disease burden, and also a wide-ranging assessment of the needs of a range of stakeholders including service providers, patients and their caretakers. In addition to the geographical location of facilities, the existence of unequal tariffs has been reported where different facilities receive between 4 GEL (2.5 USD) to 17 GEL (10.6 USD) per patient despite providing the same outpatient services. This naturally affects the resources available to provide these services.

There is currently a lack of provision for some common mental disorders, especially anxiety and OCD, as is the case in a number of European countries [15]. A comprehensive mental health system would ensure that all conditions diagnosed are included within the health care programme. There may however be an alternative route to integrate anxiety disorders with physical healthcare under the provision of the *UHCP* introduced in 2013. Moreover, the concept of public mental health, especially relating to preventative health or risk reduction, is not currently seen as a priority and currently no programmes exist to ensure the wellbeing of the population.

### Resource inappropriateness

The balanced care model highlights the need for the provision of mental health services balanced appropriately between inpatient and outpatient/community care and the management of conditions using a balance of pharmacological and psychological treatments [16]. The current mental healthcare budget allocates more than 70% of resources to hospital care, whilst less than 30% is assigned to outpatient services. Whilst there has been some diversification of services with the introduction of psychosocial rehabilitation and crisis intervention, current demand far outstrips supply. The current provision of community teams is extremely limited, with few resources allocated to this area and restrictive budgets preventing their development. Furthermore, only one supported housing service is provided in the whole of the country, with just 100 places, meaning that many patients that could be discharged from long-term care often remain in hospital due to a lack of social support, increasing the risk of institutionalisation.

Although de-institutionalisation seemed to have been the direction of travel of many FSU and post-communist countries, there has been some concern that a reduction in psychiatric beds can shift the patient burden to other sectors representing a re-institutionalisation of mental health care, as first proposed by Penrose [17]. Recent evidence has suggested that this does not seem to be the case in the majority of FSU countries [18]. Despite this, re-institutionalisation has been observed in a number of Western European countries [19].

Moreover, the *SPMH* lacks an integrated approach, which results in separate funding for the different levels of mental health care. Inpatient facilities are not involved in patient supervision after discharge. As a consequence, the continuum of care is not ensured.

Another issue that has been identified is that of the designation of all new admissions as acute care patients, for whom a higher tariff is paid than for long-term care patients. The classification of these ‘acute care patients’ is an administrative classification i.e. due to their recent admission, not based on their actual clinical need and resource requirements, which leaves the system open to manipulation. Developing the financial models that encourage providers to create and maintain a continuum between different levels of mental health services and discourage policy makers from separating budgets according to the type of care may support the development of a ‘fairer’ system.

Furthermore, the three-week time period during which an acute crisis should be resolved and the patient either discharged or transferred to long-term care, has been arbitrarily set based on financial rather than clinical need and may act as a barrier to the delivery of patient-centred care.

### Resource inflexibility

Strict public finance management rules and “silo budgeting” do not allow facility managers to appropriately re-allocate recources based on needs. This acts as a barrier to innovation and the diversification of services at a facility level. At the patient level, complications emerge due to restrictive policies that prevent an individual patient simultaneously receiving inpatient treatment from more than one programme; thus treatment covered by the *SPMH* cannot occur concurrently with treatment for a physical healthcare condition. This has a significant effect on those with co-morbidities, requiring discharge from one programme before patients are eligible for treatment within another.

### Resource timing

Knapp et al. broadly outline resource timing to include areas such as training, supply and capacity; in the context of the mental health system in Georgia, a number of barriers were identified within this domain. These barriers mainly focus on capacity within the system, both in terms of clinical care and resources within the system to plan and monitor the delivery of health services. The low numbers of psychiatrists, especially those in training, and the lack of specialist mental health nurses were identified as major challenges to the health system, both currently and for the future. Whilst there are currently only 3.92 psychiatrists per 100,000 people, which is higher than the median for lower middle income countries (0.54 per 100,000), this number falls significantly short of the European median of 8.59 per 100,000 [5]. In addition, the limited mental health human resources are not adequately used. Psychiatrists spend inadequate amounts of time with patients, as most of their time is taken up by paper work. Shifting responsibilities between doctors and nurses and introducing electronic patient records would free up psychiatrists’ time for patients [20, 21]. With relatively low salaries and perceived poor working conditions, many medics have limited interest in pursuing a career in psychiatry. Without effective monitoring and workforce planning, this is unlikely to change. In addition to the lack of diversity of services, there is also a lack of diversity of human resources, with mental health facilities almost solely staffed by psychiatrists and general nurses. If a diversification of service provision is pursued, as is hoped by many, a concomitant diversification of the workforce is also required to ensure that the right skills and competencies are also developed.

### Limitations

The study contains the following limitations: First, the study included small sample size for patients and their caretakers, 16 participants were enrolled into the study within this subgroup. Therefore, patient level (individual) findings might be restricted for wider generalizability. Second, given the specificities and contextual factors of Georgian mental health system, the study findings should be cautiously discussed in different settings and in other geographic locations.

## Conclusion

The development of comprehensive and equitable mental health care requires a deeper understanding of these barriers, which this paper aims to present. A number of clear and achievable steps are proposed:

- Adopt new financial models that create a positive stimulus for facility managers to provide a continuum of mental health care, which should be assessed through predetermined indicators, to develop high-quality and efficient services that promote the wellbeing of patients;
- Establish an appropriate budget for outpatient mental health services that considers the current needs of patients;
- Foster innovation through a unified public drug procurement system to maintain the quality of all medicines; and
- Develop a new workforce strategy to ensure the recruitment and retention of all healthcare professionals in the mental health field, and the efficient use of current health workforce resources, including task shifting from higher-trained health workers to less highly trained personnel.

The future value of the mental health system in Georgia is doubtful unless adequate resourcing is provided and the system-wide economic barriers outlined here are addressed. This includes building the capacity for leadership and stewardship at the MoLHSA and improved used of data to monitor and evaluate services and make decisions and policies based on local and international evidence.

## Additional file

Additional file 1: Survey guide developed by the research team as a basic document for different type of interview and focus group discussion guides preparation. (DOCX 30 kb)

## Abbreviations

CIS: Commonwealth of Independent States
DALY: Disability-Adjusted Life Years
FGD: Focus Groups Discussion
FSU: Former Soviet Union
GDP: Gross Domestic Product
GEL: Georgian Lari
HFADB: European Health for All database
HIC: High Income Countries
LMC: Lower Middle income Countries
LMIC: Low and Middle Income Countries
MoLHSA: Ministry of Labour, Health and Social Affairs
NGO: Non-Governmental Organization
OCD: Obsessive Compulsive Disorder
PPP: Purchasing Power Parity
SPMH: State Programme for Mental Health
UHCP: Universal Health Coverage Programme
UMC: Upper Middle income Countries
USD: United States Dollar
WHO: World Health Organization
