# Supplementary material for: Barriers to delivering mental health services in Georgia with an economic and financial focus: informing policy and acting on evidence
Source: BMC Health Serv Res. 2018 Feb 13;18:108. doi: 10.1186/s12913-018-2912-5 (PMC5809973; doi:10.1186/s12913-018-2912-5)
Supplement: Supplementary file 1 — Survey guide developed by the research team as a basic document for different type of interview and focus group discussion guides preparation. (DOCX 30 kb) [file 12913_2018_2912_MOESM1_ESM.docx]

**Economic and Financial Analysis of Mental Health System in Georgia – Survey guide**

**Section 1 – Inpatient Services**

**Desk-based work**

It would be helpful to c**omplete Table 1 which helps provide an overview of the main types of mental health inpatient care facilities in Georgia**. For instance, this will help give a sense of whether most of this care is provided in stand-alone psychiatric facilities, within general hospitals or in other facilities. In the table below, it would be helpful if we can provide a brief overview of the location of mental health beds in Georgia, e.g. the towns where psychiatric hospitals and general hospitals with psychiatric beds are found. Can we easily get information on the number of psychiatric beds in these different types of facility? Helpful if we can.

Is it also possible to get any data on average length of stay in these inpatient facilities?

In terms of how services are paid do we know if contracts or other regulatory devices influence the financing mechanisms used to pay for services in facilities? For instance, are there limits on the amount of activities that are funded by specific payment mechanisms?

In term of how services are organised do patients (and their families) have any choice over the inpatient facilities they use?

**Overview of inpatient service providers**

| **Provider Type** | **Location**  Name and location of major facilities if possible. | **Number of beds**  If possible provide your best estimate of the total number of beds available in each type of facility? E.g. 100 beds in standalone acute care only psychiatric facilities; 45 in standalone long-term care only (more than six months) psychiatric facilities etc; If possible to provide an even more detailed breakdown for some of the named major facilities this would be helpful. | **Legal Status**  Please provide an indication of the legal status of these facilities: for instance, are they owned by government but operating as independent companies, operating as private not-for-profit organisations or as private for-profit organisations. | **Who pays? Who is the contractor of the service?**  Please state the name(s) and legal status of organisations that pay for psychiatric inpatient care either at country or regional level as appropriate. | **Payment mechanisms used to pay the provider (specify % of total sources of finance or level of importance)**  **BUDGET** for Fixed Time Period  **DAY FEE =** Fixed Fee per day spent in hospital  **FEE FOR SERVICE =** Fixed Fee for each service provided  **DRG (Diagnosis Related Group)** Payment = Defined level of reimbursement for treating specific diagnosis or cluster of symptoms |
| --- | --- | --- | --- | --- | --- |
| Stand-alone psychiatric facility– acute care only |  |  |  |  |  |
| Stand-alone psychiatric facility – long-term care (length of stay over 6 months) only |  |  |  |  |  |
| Stand-alone psychiatric facility (long-term and acute care) |  |  |  |  |  |
| Psychiatric departments in general hospitals |  |  |  |  |  |
| Other, please describe |  |  |  |  |  |

**Interviews**

This set of questions and topics is meant to act as a guide for interviews with stakeholders working in acute and long stay facilities. It is intended to be flexible and interviewers should ask appropriate supplemental questions at their discretion. Not all of these questions may be appropriate for all interviewees.

1. **Sources of funding**
2. What percentage of your total funding comes from the Social Service Agency budget?
3. If you receive any additional funding from other sources for the inpatient mental health services that you provide, please briefly describe. For instance, this could include donor aid, payments from other government departments, e.g. from the Ministry of Justice for some forensic patients, from local government, private health insurance companies or from the universal health programme.
4. Is there a contract with the Social Services Agency for the provision of services and if so, how detailed are these contracts? For instance, do they have requirements on the level of different types of activity that the facility must offer? Is payment linked in any way to performance or measurement of quality?
5. **Reimbursement mechanisms**
6. Do you receive a defined budget to provide inpatient services? In the case of budgets please briefly explain how these are normally set, e.g. based on historical allocations of resources, based on maintaining specific rates of bed occupancy, adjustments for population need including age and past activity in terms of severity of cases treated etc. Please indicate if any unspent money can be retained at the end of the financial year.
7. Do you charge fees to the Social Services Agency (or any other funder) for each day a patient spends in your facility? If so please briefly describe the fee structure and indicate if there is a maximum fee level or if these fees change depending on length of stay?
8. Please describe reimbursement and reporting mechanisms with SSA.
9. Do you charge fees to the Social Services Agency (or any other funder) for each individual service that a patient is received in your facility? If so please briefly describe the fee structure and indicate if there is a maximum fee level that can be reimbursed by the Social Services Agency?
10. Do you make use of specific diagnosis or symptom related fees to charge the Social Services Agency (or any other funder) for services in your facility? If so please briefly describe the fee structure and indicate if there are any adjustments to these fees to take account of differences in length of stay or because of additional physical or mental health problems? If not linked to diagnosis, please explain how the system works. Please give an indication of whether tariffs are set at national, regional or other level. Please indicate if tariffs vary depending on co-morbidity.
11. **Budget spending**
12. Have you ever had deficit / proficit? If yes, when it happened last time? And what did you do to solve that problem?
13. **User charges**
14. Do inpatients have to make any payments for any drugs received? If so, please describe.
15. Are there any other user charges that patients have to pay for inpatient services? If there are charges do they, vary by type of ward or diagnosis? If there are charges are there any ceilings on total charges?
16. If there are charges are any specific groups of the population exempt from user charges? Are family members liable to pay any charges if the service user cannot pay?
17. If there are charges can you provide examples of the typical user charges for stays in psychiatric inpatient care services? For instance, are there daily charges for each day of a stay or a fixed charge per stay in the facility. Do patients have to pay for meals? If inpatients receive a public pension or disability benefit can the hospital take these benefits?
18. Please indicate if there are any significant additional ‘under the table’ or informal payments made to hospitals for inpatient care.
19. **Financial incentives and disincentives**
20. We would like to know something about the way in which the financing system influences the type of services that are provided in your facility.
21. Does the financing system mean that inpatients might be discharged too quickly or spend too long in hospital? If so please explain.
22. Does the financing system affect the rate of readmission? If so please explain
23. Do you receive funding to cover additional physical health needs of your inpatients e.g. management of diabetes? If so please explain
24. Does the financial system mean that some groups of patients are more or less likely to receive care in your hospital? For instance, are there any incentives which mean that your service is more likely to deal with individuals with specific types of diagnoses such as psychosis or severe depression?
25. Please describe any other important financial and/or non-financial incentives or disincentives that might encourage/discourage the use of psychiatric inpatient care service instead of the different outpatient services.
26. Please describe any other important financial and/or non-financial incentives or disincentives that influence the provision and use of inpatient services.
27. **Opportunities for reform**
28. In your opinion what are the key challenges that the mental health system in Georgia currently has to face?
29. What are the key steps that you would like to see taken to improve the organisation and quality of the overall mental health system?
30. What are the key steps that you would like to see taken to improve the organisation and quality of inpatient mental health services?

**Section 2 – Outpatient Services**

**Desk-based work**

It would be helpful to c**omplete Table below which helps provide an overview of the main types of psychiatric outpatient care facilities in Georgia**. For instance, this will help give a sense of whether most of this care is provided in stand-alone outpatient centres, dispensaries, at general hospitals or in other facilities. A key issue will be the way in which outpatient services are paid for.

In term of how services are organised do patients (and their families) have any choice over the outpatient facilities they use?

**Overview of outpatient service providers**

| **Provider Type** | **Location**  Is it possible to get a sense of how widely available these different outpatient services are? Can we easily identify how many there are or the geographical spread? | **Legal Status**  Please provide an indication of the legal status of these outpatient facilities: for instance, are they owned by government but operating as independent companies, operating as private not-for-profit organisations or as private for-profit organisations. | **Who pays? Who is the contractor of the service?**  Please provide an indication of the legal status of these outpatient services: for instance, are they owned by government but operating as independent companies, operating as private not-for-profit organisations or as private for-profit organisations. | **Payment mechanisms**  **(specify % of total sources of finance or level of importance)**  **BUD=**Budget  **FFS**=Fee For Service  **DRG (Diagnosis Related Group)** Payment = Defined level of reimbursement for treating specific diagnosis or cluster of symptom  **OTH:** please specify |
| --- | --- | --- | --- | --- |
| Dispensaries |  |  |  |  |
| Policlinics |  |  |  |  |
| Outpatient service of a psychiatric hospital |  |  |  |  |
| Psychiatric outpatient services of a general hospital |  |  |  |  |
| Other, please describe |  |  |  |  |

**Interviews**

This set of questions and topics is meant to act as a guide for interviews with stakeholders working in different outpatient facilities. It is intended to be flexible and interviewers should ask appropriate supplemental questions and follow up responses from interviewees at their discretion. The aim is to get as much insight as we can on how financing and funding arrangement impact on the services delivered and the outcomes for patients. Not all of these questions may be appropriate for all interviewees depending on their responses to initial questions.

1. **Sources of funding**
2. What percentage of your total funding comes from the Social Service Agency budget?
3. If you receive any additional funding for the mental health services from other sources could you please briefly describe? For instance, this could include donor aid, payments from other government departments, local government, private health insurance companies or from the universal health programme.
4. Is there a contract with the Social Services Agency for the provision of outpatient services and if so, how detailed are these contracts? For instance, do they have requirements on the level of different types of activity that the outpatient facility must offer? Is payment of the budget linked in any way to performance or measurement of quality?
5. **Reimbursement mechanisms**
6. Do you receive a defined budget to provide outpatient services? In the case of budgets please briefly explain how these are normally set, e.g. based on historical allocations of resources or capitation with a set budget received linked to a defined population, with adjustments for population need including age and past activity etc. Please indicate if any unspent money can be retained at the end of the financial year.
7. Do you charge fees to the Social Services Agency (or any other funder) for each time a patient comes to your outpatient facility? Do fees differ by diagnoses?
8. Please describe reimbursement and reporting mechanisms with SSA.
9. Do you make use of specific diagnosis or symptom related fees to charge the Social Services Agency (or any other funder) for services in your outpatient facility? If so please briefly describe the fee structure and indicate if there are any adjustments to these fees to take account of differences in length of stay or because of additional physical or mental health problems? If not linked to diagnosis, please explain how the system works. Please give an indication of whether tariffs are set at national, regional or other level. Please indicate if tariffs vary depending on co-morbidity.
10. **Budget spending**
11. How do you distribute global budget for outpatient services among budget items?
12. Have you ever had deficit / proficit? If yes, when it happened last time? And what did you do to solve that problem?
13. **User charges**
14. Do outpatients have to make any payments for any drugs received in your outpatient facility? If so, please describe.
15. Are there any other user charges that outpatients have to pay for? Are there any limits on total charges?
16. If there are charges are any specific groups of the population exempt from user charges?
17. Are family members liable to pay any charges if the service user cannot pay?
18. **Financial incentives and disincentives**
19. We would like to know something about the way in which the financing system influences the type of outpatient services that you provide.
20. Does the financing system mean that some outpatients may be discharged too quickly or spend too long in hospital? If so please explain.
21. Does the outpatient financing system affect the rate of readmission to inpatient services? If so please explain
22. Do you receive funding to cover additional physical health needs of your outpatients? If so please explain
23. Does the financial system mean that some groups of patients are more or less likely to receive care at your outpatient service? For instance, are there any incentives which mean that your service is more likely to deal with individuals with specific types of diagnoses e.g. psychoses versus depression?
24. Please describe any other important financial and/or non-financial incentives or disincentives that might encourage/discourage the use of psychiatric outpatient care service instead of the different inpatient services.
25. Please describe any other important financial and/or non-financial incentives or disincentives that influence the provision and use of outpatient services.
26. Are there any differences in salaries of doctors between state-funded and self-funded patients?
27. If yes, are there any motivations for doctors to manipulate with diagnoses?
28. **HR**
29. How do you think, is there enough HR to meet the requirements of patients in your facility? In general?
30. How frequently are the psychiatrists involved in trainings?
31. **Opportunities for reform**
32. In your opinion what are the key challenges that the mental health system in Georgia currently has to face?
33. What are the key steps that you would like to see taken to improve the organisation and quality of the overall mental health system?
34. What are the key steps that you would like to see taken to improve the organisation and quality of outpatient mental health services?
